# Myalgic Encephalomyelitis/Chronic Fatigue Syndrome: The Human Herpesviruses Are Back!

**DOI:** 10.3390/biom11020185

**Published:** 2021-01-29

**Authors:** Maria Eugenia Ariza

**Affiliations:** 1 Department of Cancer Biology and Genetics, The Ohio State University Wexner Medical Center, Columbus, OH 43210, USA; maria.ariza@osumc.edu; 2 Institute for Behavioral Medicine Research, The Ohio State University Wexner Medical Center, Columbus, OH 43210, USA

**Keywords:** myalgic encephalomyelitis/chronic fatigue syndrome, epstein-barr virus (EBV), human herpesvirus 6 (HHV-6), immune dysfunction, BRRF1, BLLF3, deoxyuridine triphosphate nucleotidohydrolase (dUTPase)

## Abstract

Myalgic Encephalomyelitis/Chronic Fatigue Syndrome (ME/CFS) or Systemic Exertion Intolerance Disease (SEID) is a chronic multisystem illness of unconfirmed etiology. There are currently no biomarkers and/or signatures available to assist in the diagnosis of the syndrome and while numerous mechanisms have been hypothesized to explain the pathology of ME/CFS, the triggers and/or drivers remain unknown. Initial studies suggested a potential role of the human herpesviruses especially Epstein-Barr virus (EBV) in the disease process but inconsistent and conflicting data led to the erroneous suggestion that these viruses had no role in the syndrome. New studies using more advanced approaches have now demonstrated that specific proteins encoded by EBV could contribute to the immune and neurological abnormalities exhibited by a subgroup of patients with ME/CFS. Elucidating the role of these herpesvirus proteins in ME/CFS may lead to the identification of specific biomarkers and the development of novel therapeutics.

## 1. Introduction

Myalgic Encephalomyelitis/Chronic Fatigue Syndrome (ME/CFS) or Systemic Exertion Intolerance Disease (SEID) is a complex chronic multisystem illness of unconfirmed etiology. ME/CFS is a highly debilitating disorder characterized by severe exacerbation of fatigue and other symptoms after even mild exertion. The Centers for Disease Control (CDC)/Fukuda [1], Canadian [2], International Consensus Criteria [3] and the Institute of Medicine (IOM) [4] have each established criteria to be used in the diagnosis of ME/CFS. As such, numerous criteria, primarily long-lasting fatigue, have been used over the years to identify patients afflicted with this syndrome. The lack of a universally accepted clinical criteria has led to multiple problems and confusion as to how to accurately diagnose and stratify patients with this syndrome and has made it difficult, if not impossible, to be able to compare study outcomes in a biologically meaningful way across the board. Furthermore, numerous studies using small size patient cohorts, which lack statistical power to achieve reproducible and rigorous results, have further complicated the daunting task of identifying biomarkers/signatures that would be useful for diagnosis and stratification. These problems have resulted in conflicting data further delaying the pursuit of valid studies on triggers of this syndrome. Diagnosis of ME/CFS, which is currently conducted according to the modified Fukuda/Canadian and/or the Institute of Medicine case definition [1,2,4], is based only on symptom-based exclusion criteria, which include unexplained and persistent post-exertional fatigue accompanied by numerous symptoms related to immune, endocrine and cognitive dysfunction. The general consensus of the clinical and research community is that ME/CFS is not a single illness but rather it represents a heterogeneous population exhibiting the same symptomology, but with multiple etiologies.

## 2. Hypotheses Regarding Mechanisms of Underlying Causes of ME/CFS

**Figure 1:**
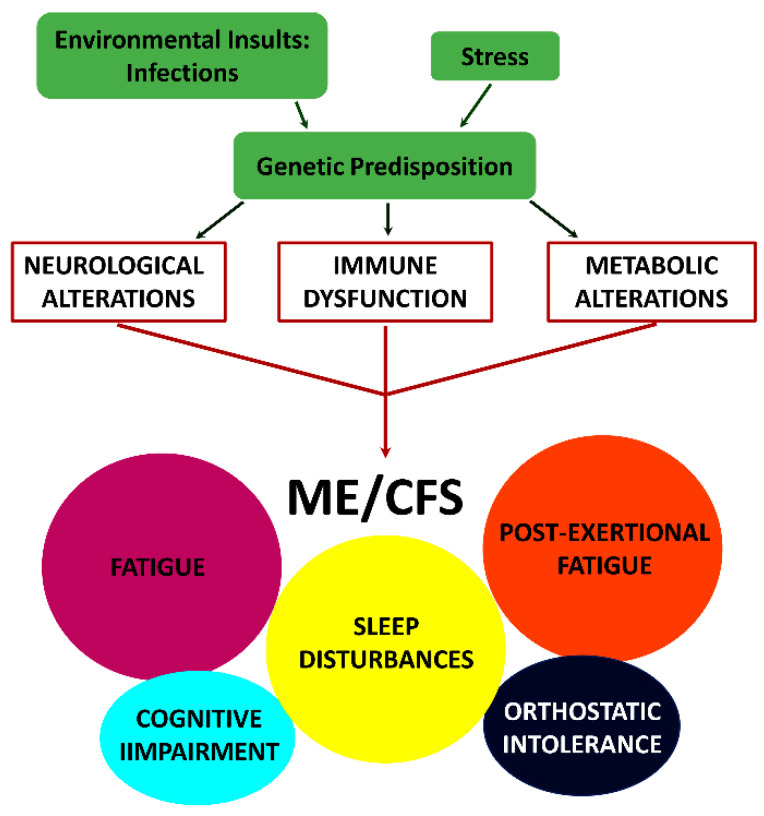
Model depicting how the combined effect of environmental insults, and stress in genetically susceptible individuals can trigger neurological, immune and metabolic dysfunction, which together could contribute to the symptomology observed in ME/CFS [4].

In addition to the new name of SEID, a new-case-criteria for clinical diagnosis was introduced in 2015 (IOM) [4]. This criteria are based exclusively upon symptoms and require that a patient has: (i) a “substantial reduction/impairment in the ability to engage in pre-illness levels of occupational, educational, social or personal activities that persists for more than six months and is accompanied by fatigue, which is new or of definite onset (not lifelong), is not the result of ongoing excessive exertion and is not substantially alleviated by rest, (ii) post-exertional malaise, (iii) unrefreshing sleep and (iv) either cognitive impairment or orthostatic intolerance.” While it is apparent that such diverse symptomology must be due to alterations in multiple physiological processes, thus a multisystem illness, the sequence of events leading to the initiation and/or progression of this disease are unknown. A model depicting the mechanism by which the combined effect of environmental insults, and stress in genetically susceptible individuals can trigger the symptomology observed in patients with ME/CFS is shown in Figure 1.

Numerous mechanisms including chronic infections, energy metabolic defects, endocrine, immunological and neurological disturbances, as well as autoimmunity have been hypothesized to explain the pathology of ME/CFS, but as of this time the etiology of ME/CFS is unknown. Many studies have been conducted that support or dispute the various hypotheses and while there are several reasons for the occurrence of such discrepancies, the bottom line is that we do not know what triggers ME/CFS or what the underlying mechanisms are which result in the manifestation of specific symptoms. 

There have been multiple reports of “outbreaks”, associated with exposure to infectious agents, as likely candidates responsible for the symptoms observed in ME/CFS patients [5]. Various bacteria, including members of the gut microbiome, and viruses such as human parvovirus B19, enteroviruses, as well as the herpesviruses Epstein-Barr virus (EBV), human herpesvirus-6 types A and B (HHV-6), and human cytomegalovirus (HCMV), have been implicated as possible triggers of ME/CFS. However, conflicting results have been obtained and the potential role of virus infections remain uncertain despite the fact that 49 to 93% of patients who develop ME/CFS report that their symptoms developed after an illness exhibiting “flu-like” symptoms [6,7,8,9]. These conflicting results are due to many variables such as heterogeneity of the patient population, the small size of patient cohorts used in some studies that does not allow for statistical evaluation, inclusion of patients who do not fit the case criteria definitions, inadequate/poor controls, different methodological approaches as well as misinterpretation of the data to name a few. 

## 3. Herpesviruses and ME/CFS

### 3.1. Serology

There are nine herpesviruses known to infect humans. These include EBV, herpes simplex virus types 1 and 2 (HSV-1 & -2), HCMV, HHV-6, human herpesvirus-7 (HHV-7), human herpesvirus-8 (HHV-8) and varicella-zoster virus (VZV). While these viruses can be distinguished based upon cellular tropism, serological reactivities, epidemiological features and DNA sequences, they share one common feature, in that all establish latent (persistent) infections in their host and can be periodically reactivated over a person’s lifetime. Herpesviruses are ubiquitous in the adult population with greater that 95% of the adults being persistently infected with EBV, HHV-6 and HHV-7.

While there have been sporadic reports in the literature concerning the potential role of HSV-1&2, HCMV, HHV-7, HHV-8 and VZV in ME/CFS such reports have not been validated. There have been also numerous studies attempting to link EBV and HHV-6 as potential triggers of ME/CFS. However, these studies provided conflicting data and resulted in Soto and Straus prematurely declaring that the evidence for herpesviruses involvement in ME/CFS was fading [10]. What are the reasons for this conclusion? Most of the initial studies linking ME/CFS to EBV were serological studies performed using the classical EBV antigens: Early antigen-diffuse pattern (EA-D), Early antigen restricted pattern (EA-R), viral capsid antigen (VCA) and Epstein-Barr Nuclear Antigen 1 (EBNA 1) [6,11,12,13,14,15,16,17,18,19,20]. Overall, these studies gave conflicting results. Recent studies using more advanced technologies such as peptide microarray [21] and suspension multiplex immunoassay [22] reported that the EBV anti-IgG response in ME/CFS patients was not significantly different than controls. In both studies there were small non-significant differences in IgG response to Epstein-Barr Nuclear antigen 3C (EBNA-3; EBNA 6) and in the Blomberg study to EA-D. These findings are consistent with numerous reports raising awareness to the fact that analyses of EBV serological data are complicated and thus, cautious interpretation is required. This is due to several factors including, but not limited to, the heterogeneity of the populations under study, the lack of case study definition, questions concerning the reliability and precision of the results obtained from different commercial laboratories as well as the lack of a demonstrated correlation between serological data and viral load indicating that classical serological data may be of limited use as an indicator of EBV reactivation [23].

### 3.2. Viral Load

With the exception of a single study by Shikova et al. [24] indicating an active EBV infection in some ME/CFS patients, most studies have reported no increase in EBV and/or HHV-6 viral load in patients with ME/CFS [20,25,26,27]. The overall result of these studies was that no significant increase in virus load occurred in ME/CFS patients when compared to controls. These findings combined with the lack of a significant serological response support the premise of Soto and Straus [10] declaring that the evidence for herpesviruses involvement in ME/CFS was “fading”. As discussed above, the inability to demonstrate a herpesvirus such as EBV or HHV-6 as triggers for ME/CFS could be due to numerous factors including the heterogeneity of the patient population, the small size of patients used in some studies, the use of patients who do not fit the case criteria definitions, and inadequate/poor controls. However, and perhaps more importantly, it might be due to the choice of methods used to approach the problem and the established dogma regarding how these viruses replicate and are maintained in vivo. 

## 4. “New” Data Suggesting That Some Herpesviruses, Particularly EBV and HHV-6, May Be Involved with the Symptomology of ME/CFS in a Subgroup of Patients

### 4.1. Abortive Lytic Replication (ALR)

A central concept regarding the biology of herpesviruses is that two distinct phases of viral gene expression exist either in latency or lytic replication where virus progeny are produced. However, there is accumulating data to suggest that, at least in the case of EBV, this central concept may be incorrect and that in vivo a third state exists: abortive lytic/leaky replication. Laichalk and Thorley-Lawson [28] first described ALR in tonsillar plasma cells and this was further supported by studies of Al Tabaa et al. [29,30], which demonstrated in plasma cells that only 10–20% of the cells expressing BZLF1 (immediate early protein that initiates lytic replication) synthesize gp350/gp220 (BLLF1 late gene expression), suggesting lack of completion of virus replication. Studies by Altmann and Hammerschmidt [31] and Wen et al. [32] demonstrated the transient expression of several immediate early and early genes following primary infection of B cells and Shannon-Lowe et al. [33] demonstrated that this also occurred in epithelial cells. Additional studies by Kalla et al. [34] demonstrated that in primary B cells, transient expression of these immediate early/early genes was required to establish latency. However, no late gene expression occurs during this time and as such this represents ALR. The transient expression of the immediate early/early lytic genes during this pre-latent state, which can last approximately fourteen days, is required for the establishment of latency in memory B cells. 

There is substantial evidence demonstrating that abortive lytic replication also occurs in EBV positive malignant tissues from primary biopsies of patients with nasopharyngeal carcinoma (NPC) [35,36,37,38], Burkitt’s lymphoma (BL) [39] and gastric carcinoma [40,41]. Furthermore, studies using a humanized mouse model demonstrated that abortive lytic replication contributed to lymphoma development in these animals [42,43]. 

Recent studies analyzing the EBV genome from T and NK cells of patients with chronic active EBV infection (CAEBV), classified as a lymphoproliferative disorder by the 2016 World Health Organization, revealed viral genomes harboring intragenic deletions in the BART region and in essential lytic genes (*BALF5*‒DNA polymerase, *BMRF1*—Double stranded DNA binding protein, *BSLF1*—primase, *BBLF2/3*—helicase-primase and *BBLF4*—helicase) [44,45]. Using a BALF5 knockout virus these investigators demonstrated that the knockout virus immortalized primary B cells as effectively as wild-type virus. Somewhat surprising was the observation that B cells immortalized with the knockout virus exhibited higher levels of lytic gene expression than cells immortalized with wild-type virus. The investigators proposed that deletions in essential lytic genes allowed reactivation of the lytic cycle but averted virus production and cell lysis. They concluded that the increased expression of viral lytic genes is reminiscent of the “pre-latent abortive lytic” state, in which a substantial number of lytic genes are produced for weeks in the absence of progeny production, which contributes to cell survival upon de novo infection. Furthermore, these results provide evidence that execution of the entire lytic program is not needed for cell growth of EBV-associated lymphomas. Interestingly, in some patients with CAEBV hypersensitivity to mosquito bites and skin lesions resembling hydroa vacciniforme, it was reported that EBV reactivation occurs in cutaneous lesions of systemic hydroa vacciniforme, but it is abortive [46]. 

Prusty et al. [47] using an epithelial (U2OS) cell culture-HHV-6 latency model recently identified an early stage of HHV-6 reactivation, termed transactivation, characterized by the transcription of several viral small non-coding RNAs (sncRNAs) and the absence of increased viral replication. The data suggest that ALR might be occurring in HHV-6 infections. Furthermore, while the lack of complete/productive viral replication occurred in these cells, the cells gained partial function by viral genome transactivation and the investigators suggested that this might have clinical significance.

Halpin et al. [48] reported a significant increase in the levels of anti-herpesviruses deoxyuridine triphosphate nucleotidohydrolase (dUTPase) antibodies in longitudinal and single sera samples from patients diagnosed with ME/CFS compared to the controls. Moreover, this group found that a significant percentage of patients with ME/CFS (30.91–52.7%) were simultaneously producing antibodies against multiple human herpesviruses (EBV and HHV-6) dUTPases and/or the human dUTPase compared to controls (17.21%). Since the herpesvirus dUTPases are only expressed during lytic/abortive lytic replication and these ME/CFS patients had no reported evidence of symptomatic herpesvirus infections, these results are highly suggestive that ALR was occurring.

### 4.2. EBV and the Immune System in ME/CFS

Loebel et al. [26] reported a significant reduction of EBNA-1 and VCA antibody secreting EBV-specific memory B cells in two separate cohorts of ME/CFS patients, who were diagnosed using the Fukuda criteria. Analyses of CD4^+^ and CD8^+^ T cell subsets in a small cohort of patients also revealed significantly diminished frequencies of EBNA-1-specific triple TNF-α/IFN-γ/IL-2 producing cells in these patients. Furthermore, when examining EBV load in PBMCs from ME/CFS patients and healthy control individuals, a higher frequency of EBER-DNA but not BZLF-1 RNA was observed in ME/CFS patients compared to healthy individuals, which the authors concluded suggested that latent replication was a frequent event. Altogether the authors concluded that their data strongly suggested a deficient EBV-specific B- and T-cell memory response in ME/CFS patients and highlighted an impaired ability to control early steps of EBV reactivation in these patients. In a follow up study using a serological peptide microarray approach they showed that the EBV anti-IgG response to peptides from proteins expressed during lytic replication (BALF2, BLRF2, BMRF1, BALF5, BZLF1, BFRF3, BLLF1, BLLF3) and during latency (EBNA1, EBNA3A, EBNA3C, LMP1, LMP2) was not significantly different from the controls. However, there were small non-significant differences in IgG response to Epstein-Barr nuclear antigen 3C (EBNA3C; EBNA 6) [21]. This was recently confirmed in a study conducted by Blomberg et al. [22] using high-throughput serological approaches. 

Several studies [49,50,51,52] by the same group showed that regulatory T cells (FOXP3^+^, Tregs) were significantly elevated in ME/CFS patients compared to controls. Tregs are a specialized subpopulation of T cells that function to suppress the immune response and, thus, their increase in ME/CFS patients could indicate an overall disruption of the immune system. Sepulveda et al. [53] recently proposed a model by which the herpesviruses may drive ME/CFS through Tregs. However, to date there is no experimental data to validate this model or to demonstrate how the herpesviruses may modulate Treg proliferation in patients with ME/CFS.

### 4.3. Can EBV Proteins Produced during ALR Contribute to the Symptomology of ME/CFS?

**Figure 2:**
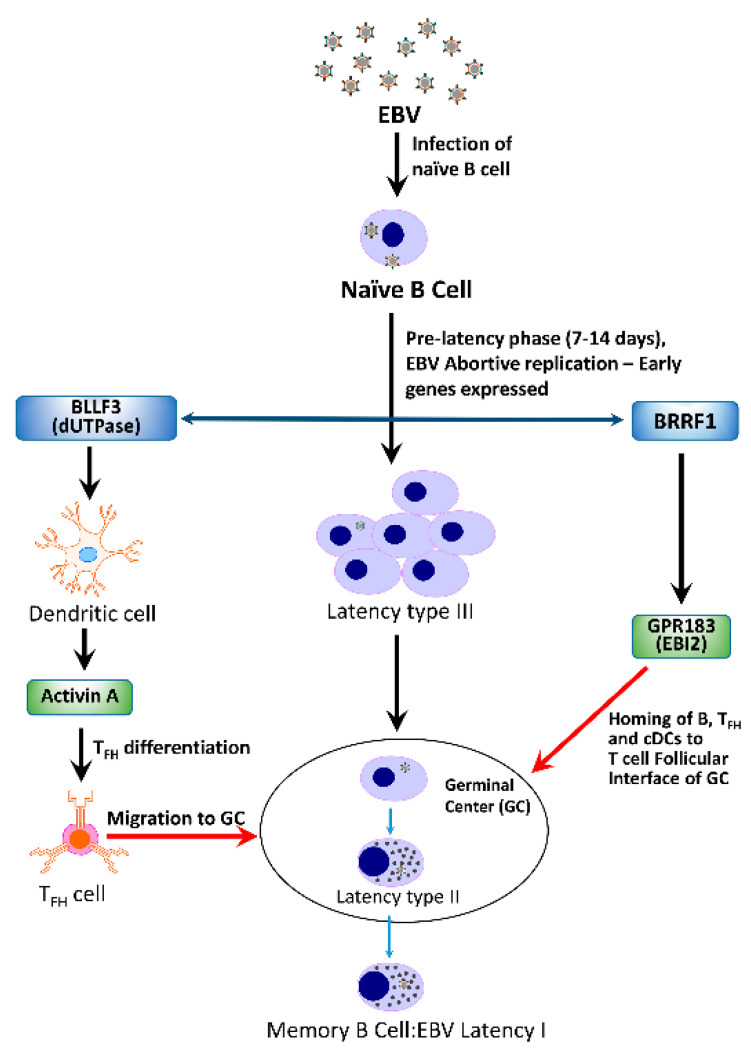
Hypothetical model depicting potential interactions between the EBV early proteins Na and dUTPase, encoded by *BRRF1* and *BLLF3*, respectively, and their contribution to the immune dysfunction observed in patients with ME/CFS.

A basic question that needs to be addressed is “If a herpesvirus is capable of triggering the development of ME/CFS in a cohort of patients, what is the mechanism(s) by which this may be accomplished?”. Could a single protein or a group of proteins be required? How does it proceed—in a linear fashion where disruption of one system results in the sequential disruption of other systems or rather in a cascade of events resulting in dysfunction of multiple systems simultaneously? A hypothetical model depicting the potential interactions between the EBV early proteins Na and dUTPase, encoded by *BRRF1* and *BLLF3*, respectively, and their contribution to the immune dysfunction observed in ME/CFS patients is shown in Figure 2. 

#### 4.3.1. *BRRF1* and Epstein-Barr Induced Gene 2 (*EBI2*)

*BRRF1* encodes for the transcription factor Na, is expressed as an early gene during lytic replication [54] and it is thought it may play a role in the switch between latent and lytic EBV replication [55]. Cornaby et al. [56] reported that the EBV *BRRF1* gene exhibited a similar expression pattern to the cellular gene Epstein-Barr induced gene 2 (*EBI2*) and that while a BRRF1-deficient virus could not induce EBI2, B cells transduced with the *BRRF1* gene resulted in up-regulation of EBI2. Using microarray gene profiling approaches, Kerr and co-investigators [57,58,59,60] identified 88 genes in PBMCs from patients with ME/CFS, which clustered into eight gene expression subtypes. Interestingly, 12 human genes (*NFKB1, EGR1, ETS1, GABPA, CREBBP, CXCR4, EBI2, HIF1A, JAK1, IL6R, IL7R and PIK3R1*) have been shown to be upregulated, either directly or indirectly, by EBV infection. Of these genes, *EBI2* was the most highly induced gene in one subgroup of patients consisting of only ME/CFS females, who had the most severe clinical phenotype, the lowest functional level on the patient short health survey form SF-36 scoring and a high frequency of muscle pain and sleep problems. *EBI2* encodes for GPR183, a member of the rhodopsin-like subfamily of seven transmembrane G protein-coupled receptors. The natural ligand of EBI2 is an oxysterol, 7α, 25-dihydroxycholesterol (7α, 25-OHC), which is expressed primarily in secondary lymph tissue by stromal cells. EBI2 regulates B cell positioning in lymphoid tissue and is necessary for initiating T cell-dependent antibody responses [61]. It has been reported that the EBI2 signaling pathway responds to proinflammatory signals and that it attenuates the response of astrocytes to proinflammatory signals in the brain [62,63]. While these results suggest that EBI2 may play a role in EBV infections, its precise role in this process remains unknown. Similarly, while EBI2 up-regulation in ME/CFS patients could possibly contribute to some immune dysfunction observed in these patients, its contributions to other symptoms such as POTS, brain fog and fatigue, need to be determined.

#### 4.3.2. BLLF3

*BLLF3* is expressed as an early gene during lytic replication of EBV and encodes for a dUTPase. dUTPases represent a family of metalloenzymes that catalyze the hydrolysis of dUTP to dUMP and pyrophosphate, thus preventing dUTP from being incorporated into DNA by DNA polymerases. Interestingly, dUTPases are encoded by numerous viruses including the human herpesviruses, vaccinia virus, African swine fever virus and human endogenous retrovirus K (HERV-K). In addition to their role in DNA synthesis, several studies have now demonstrated that the herpesviruses and HERV-K dUTPases have novel functions and are capable of altering physiological processes independent of their enzymatic activities [48,64,65,66,67,68,69,70,71,72,73,74,75,76,77,78,79]. 

##### BLLF3 in ME/CFS

It has been reported that patients with ME/CFS exhibit a statistically significant increase in anti-EBV/HHV-6 dUTPase antibodies compared to controls [48,70]. This suggests that expression of the *BLLF3* gene of EBV and the U45 gene of HHV-6 is occurring in this subgroup of patients with ME/CFS and raises the question could the dUTPase proteins be contributing to the symptomology observed in these patients?

##### BLLF3 Pro-Inflammatory Cytokines and Immune Dysfunction

There have been numerous studies addressing cytokines and/or cytokines networks in patients with ME/CFS with the hope of elucidating possible mechanisms that cause immune dysfunction and also of finding a “signature” that would be useful in diagnosing or stratifying symptom severity [49,80,81,82,83]. Systematic reviews of the literature coupled with a few meta-analysis studies have produced conflicting results [84,85,86,87]. TGF-β was identified in one study as the only cytokine elevated in ME/CFS patients [84] while another study reported that TNF, IL-2, IL-4 and C-reactive protein were elevated in ME/CFS patients [85]. Yet two additional studies concluded that there were no significant differences in the levels of plasma cytokines in ME/CFS patients [86,87]. There are many reasons that may have contributed to the problems associated with developing a specific cytokine signature in ME/CFS, as discussed earlier, and as such these studies do not preclude the possibility that specific cytokines may contribute to the immune dysfunction observed in these patients.

Initial studies using EBV dUTPase as the prototype for the monomeric herpesviruses dUTPases demonstrated that it was capable of inducing the increased secretion of multiple cytokines and chemokines including TNF-α, TGF-α, IL-1β, IL-6, IL-8, IL-12p40, IL-23, CCL5, CCL20, and IFN-γ in PBMCs and human primary monocyte derived dendritic cells (hDCs) [67,72,74,79]. TNF-α, IL-1β, and IL-6 have been identified as important modulators of sickness behavior in mice [88]. The EBV dUTPase has been reported to cause anxiety and sickness behavior, which is enhanced by chronic stress [73,75,76]. IFN-γ has also been associated with the early phase of ME/CFS, suggesting a possible virus trigger for the disease [81].

To determine the mechanism(s) by which EBV dUTPase induced heightened cytokine and chemokine levels, studies using specific inhibitors, blocking antibodies and various dominant negative constructs demonstrated that the increase expression of these cytokines/chemokines was dependent on Toll-like receptor 2 (TLR2) signaling, which resulted in NF-κB activation [67]. Furthermore, it was demonstrated that EBV dUTPase is secreted in exosomes from chemically induced Raji cells at sufficient levels to induce NF-κB activation and cytokine secretion in hDCs and PBMCs through a TLR 2-dependent mechanism [72]. Exosomes are produced by numerous cell types, have been implicated in numerous diseases, including ME/CFS [84], and are capable of trafficking to various organs within the body, including the brain, where they function as intercellular messengers. These results demonstrated that the EBV dUTPase is acting as a novel Pathogen-Associated Molecular Pattern (PAMP) ligand protein for TLR2 [72]. Additional studies demonstrated that the ability to act as a PAMP was not a unique property of EBV dUTPase but a common feature to the dUTPases encoded by other herpesviruses (HSV-2, HHV-6A, HHV-8, VZV) and the human endogenous retrovirus-K [68,74]. However, unlike EBV dUTPase, which formed TLR2 homodimers, these dUTPases differentially activated NF-κB through ligation of TLR2/TLR1 heterodimers [74].

##### BLLF3 in Autoimmune Disease 

Several studies have demonstrated the presence of autoantibodies in patients with ME/CFS against numerous cellular components including anchorage molecules [89,90], heat shock protein 60 [91], human nuclear dUTPase [48], microtubule associated protein 2 [92], muscarinic cholinergic and β-adrenergic receptors [93,94] nuclear envelop protein lamin B1 [95], serotonin [96,97] and single and double stranded DNA [98], resulting in the hypothesis that ME/CFS may represent an autoimmune disease [99,100]. It has been suggested that EBV may be inducing the formation of autoreactive B cells through a molecular mimicry process with an EBV antigen and self-antigens [99,100]. However, studies to demonstrate whether or not EBV is inducing the formation of autoantibodies in patients with ME/CFS through a molecular mimicry mechanism are lacking [21,53]. Furthermore, studies to address B cell populations in patients with ME/CFS have provided conflicting information [51,101,102,103,104]. 

Cox et al. [105] have recently demonstrated a potential mechanism by which the EBV and HHV-6 dUTPases could contribute to autoantibody development. Examination of sera from ME/CFS patients revealed significant increases in levels of activin A and IL-21 but not CXCL13 in ME/CFS patients, which correlated with seropositivity for anti-EBV and anti-HHV-6 dUTPase antibodies in these patients. Activin A belongs to the transforming growth factor-beta (TGF-β) superfamily and is a pleiotropic cytokine affecting several cell types involved with immune regulation. Activin A has been implicated in several autoimmune and inflammatory diseases but a causal role has not been established [106]. Elevated activin A expression has been linked to muscle wasting and loss of muscle mass [107,108]. IL-21 is a pleiotropic cytokine produced primarily by invariant natural killer T cells (iNKT) cells, follicular helper T (T_FH_) cells and T_H_17 cells [109]. IL-21 is required for the differentiation of T_FH_ cells, which are important for the germinal center (GC) antibody response [110]. More importantly, increased serum IL-21 levels have been reported in patients with autoimmune diseases [111].

In a parallel study, these investigators demonstrated that EBV and HHV-6 dUTPases induced the secretion of activin A in hDCs at sufficient quantities to promote the differentiation of naïve CD4^+^ T cells into a T_FH_ cell-like phenotype [105]. Interestingly, serum from ME/CFS patients was sufficient to drive naive CD4^+^ T cell differentiation into a T_FH_ cell-like phenotype. 

In addition, immunophenotyping studies demonstrated that the EBV dUTPase protein induced a significant increase in the frequency of iNKT_FH_ cells, marginal zone B (MZB) cells and plasmablasts/plasma cells in vivo, which was supported by gene expression analyses [105]. iNKT cells provide B cell help in a cognate T cell-dependent response [111,112,113]. Such an interaction leads to the development of extrafollicular foci, abortive GC formation, low affinity maturation and short-lived plasma cells. MZB cells also differentiate into short-lived, low-affinity extrafollicular plasma cells which are important for mounting a rapid antibody response to pathogens [114,115]. If dysregulated, these processes can lead to the production of auto-reactive antibodies and autoimmunity. Altogether, these data suggest that some patients with ME/CFS may exhibit a dysfunctional GC antibody response and elevated T_FH,_ both of which have been implicated in several autoantibody-associated autoimmune diseases, including systemic lupus erythematous, lupus nephritis, rheumatoid arthritis and multiple sclerosis. Furthermore, the data support a role for EBV dUTPase protein in this process by stimulating an extrafollicular antibody response, which could result in the formation of autoreactive B cells and subsequently, the production of autoreactive antibodies. While there has been a single study conducted in ME/CFS patients that reported no differences in MZB cell frequency between case and control groups [49], several studies have shown an increase in iNKT cells in ME/CFS patients [51,52,53], which positively correlated with disease severity in some reports [51,52].

##### Neuroinflammation

Neuroinflammation is a common feature of ME/CFS, affecting 85–90% of all patients, yet the underlying mechanism(s) responsible for the initiation and/or promotion of this process is largely unknown. Although neuroimaging studies have found structural and functional alterations in the brains of ME/CFS patients, there is limited evidence to suggest activation of astrocytes and microglia or widespread neuroinflammation in the brains of these patients [116]. Despite the fact that there are in vivo studies in mice suggesting the involvement of the NLRP3 inflammasome in the neuroinflammatory process [117,118] and a study in humans indicating metabolic and temperature abnormalities in the brains of patients with ME/CFS [119], studies to demonstrate the mechanism(s) by which neuroinflammation was induced as well as the consequences of this process are lacking. 

**Figure 3:**
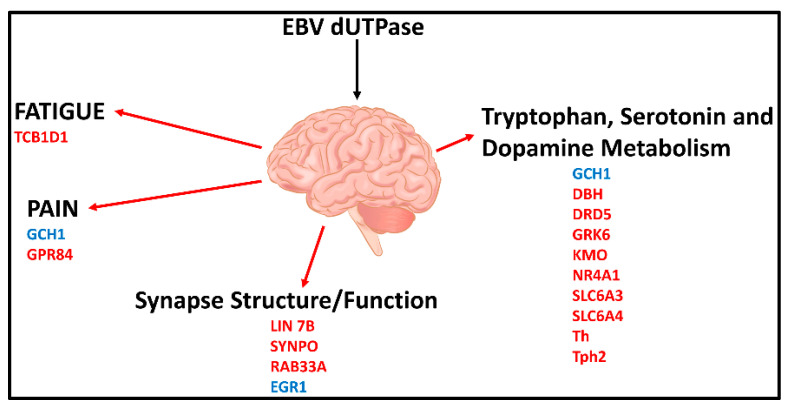
Effects of EBV dUTPase on gene expression in brains of female C57Bl/6 mice (6–8 weeks old) and the potential neurological circuits modulated with direct implications in ME/CFS. Genes in blue were significantly upregulated while those in red were significantly downregulated [120].

A recent study [120] in a mouse model revealed that EBV dUTPase altered the expression of 34 genes with central roles in blood-brain-barrier (BBB) integrity (*CGN, TJP2, RAPGEF6*), fatigue (*TCB1D1*), pain (*GCH1, GPR84*), synapse structure (*LIN 7b, SYNPO, RAB33A*) and function (*Egr1*), as well as tryptophan, dopamine and serotonin metabolism (*GCH1, DBH, DRD5, GRK6, KMO, Nr4a1, Slc6a3, SLC6a4, Th, Tph2*) (Figure 3). 

**Figure 4:**
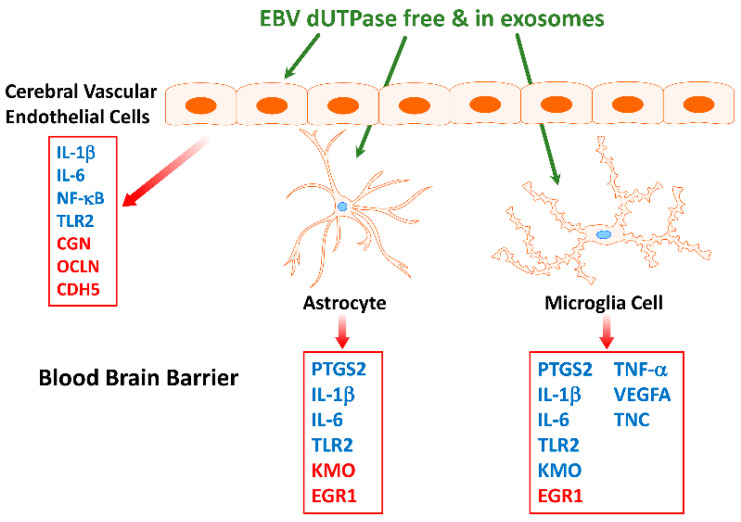
Diagram demonstrating the effect of EBV dUTPase on gene expression in immortalized primary human cerebral microvascular endothelial cells, astrocytes and microglia cells. Genes in blue were significantly upregulated while those in red were significantly downregulated when compared to controls [120].

Furthermore, EBV dUTPase may alter synaptic plasticity in vivo, which is important in learning and memory processes, as indicated by the ability of the dUTPase protein to downregulate the expression of *LIN7b, SYNPTO, and RAB33A* and upregulate *Egr-1* in mouse brain. These genes have critical functions in (1) ensuring proper localization of the GRIN2B subunit of the N-methyl-D-aspartate receptor (NMDAR), (2) long-term potentiation, (3) mediating antegrade axonal transport of post-Golgi synaptophysin-positive vesicles and their fusion at growth cones, and (4) NMDAR-mediated downregulation of PSD95 and α-amino-3-hydroxy-5-methyl-4-isoxazolepropionic acid (AMPAR) trafficking, all of which are important for synaptic development, plasticity, and functions. AMPAR and NMDAR play critical roles in the plasticity of most excitatory synapses, as indicated by a number of neurologic disorders associated with synaptic dysfunction that have altered NMDAR and AMPAR expression, trafficking, and signaling. These data suggest that the EBV dUTPase is capable of altering synaptic structure and function as well as neuronal communication, which would affect cognitive processes. In addition, in vitro studies using immortalized primary human cerebral microvascular endothelial cells (hMCEs), astrocytes and microglia the investigators demonstrated that the EBV dUTPase was a potent inducer of the proinflammatory cytokines TNF-α, IL-1β and IL-6, which are known to disrupt the BBB (Figure 4). Thus, the in vitro and in vivo data provide evidence supporting the premise that EBV dUTPase protein could disrupt the BBB, which would allow the passage of inflammatory mediators and cells, including dendritic, B and T cells into the brain.

Furthermore, while EBV dUTPase induced a transient increase in the expression of (*PTGS2/COX-2*) in human astrocytes, it induced a significant and sustained (>24 h) increase in microglia. The role of COX-2 as an important contributor to neuroinflammatory toxicity in neurodegenerative diseases is well established [121]

Finally, these studies found that the EBV dUTPase protein modulates tryptophan, serotonin, and dopamine metabolism and use in vitro and in vivo. The EBV dUTPase may alter kynurenine catabolism in microglia in vitro by increasing the expression of indoleamine 2,3 dioxygenase (*IDO1*) and kynurenine-3-monooxygenase (*KMO*), suggesting that there is an increase synthesis of quinolinic acid. Quinolinic acid, an agonist of NMDAR, can cause overstimulation that results in neuronal toxicity. Furthermore, the data suggest the EBV dUTPase protein increases the expression of GTP cyclohydrolase *(Gch1)*, and down-regulates both tryptophan hydrolase 2 (*Tph2*) and tyrosine hydrolase (*Th*). *Gch1* is the rate limiting enzyme necessary for the synthesis of tetrahydropterin (BH4), a substrate required for serotonin and dopamine synthesis by *Tph2* and *Th*, respectively. Furthermore, the dopamine receptors Drd1 and Drd5, as well as the serotonin transporter gene Slc6a4, were also down-regulated. These results suggest that the decreased synthesis and recycling of dopamine and serotonin coupled with the increased degradation by astrocytes and microglia could result in low levels of these neurotransmitters ultimately leading to cognitive defects and increased oxidative stress. The dopaminergic and serotonergic neurotransmitter systems are reported to play a critical role in the regulation of emotion and mood, and have been implicated in a wide spectrum of neuropsychiatric disorders. Specifically, EBV dUTPase simultaneously down-regulated key genes involved with dopamine and serotonin synthesis as well as key transporters and receptors required for signaling by these molecules. Low dopamine levels are associated with fatigue, attention deficits, decreased motivation and depression [122], while low serotonin levels are associated with fatigue, cognitive impairments, anxiety and digestive problems [123]. These are common symptoms associated with ME/CFS. Morris et al. [124,125] proposed that alterations in tryptophan catabolism may contribute to a variety of symptoms observed in several neuroimmune disorders including ME/CFS.

## 5. Conclusions and Future Directions

It is clear that the lack of a universally accepted clinical criteria has led to multiple discrepancies, problems and confusion as to how to accurately diagnose and stratify patients with ME/CFS. This has severely hampered the pursue of studies to clearly define the environmental and genetic factors that act as triggers or the downstream mechanisms responsible for the development/progression of ME/CFS. Furthermore, numerous studies using small size patient cohorts, which lack the statistical power to achieve reproducible and rigorous results, have further complicated the task of identifying biomarkers/signatures that would be useful for diagnosing patients. 

The role of some herpesviruses in the development and evolution of ME/CFS in a subset of patients has also been hampered because of the use of classical serological approaches focused primarily on viral proteins expressed during latency or late in the replicative cycle of these viruses or viral load as indicators for the involvement of herpesviruses in the pathobiology of ME/CFS. Recent studies using more advanced serological approaches as well as mechanistic studies have demonstrated the possible role of the EBV BRRF1 and BLLF3 gene products in ME/CFS pathophysiology. Future directions should focus on exploring the use of these gene products for the development of novel therapeutics and/or as biomarkers with diagnostic application or disease progression. Additionally, additional studies need to be performed in light of the new evidence showing high level of abortive lytic replication of these viruses to determine whether other early herpesvirus proteins could contribute to the disease process. Finally, since there is evidence suggesting simultaneous reactivation of multiple herpesviruses in a large percentage of ME/CFS patients, studies should examine whether or not there is cooperative effects between these viruses as well as other viruses within the virome that could contribute to human disease.
